# New Look on 3-Hydroxyiminoflavanone and Its Palladium(II) Complex: Crystallographic and Spectroscopic Studies, Theoretical Calculations and Cytotoxic Activity

**DOI:** 10.3390/molecules21040455

**Published:** 2016-04-13

**Authors:** Maria Kasprzak, Małgorzata Fabijańska, Lilianna Chęcińska, Leszek Szmigiero, Justyn Ochocki

**Affiliations:** 1 Department of Bioinorganic Chemistry, Medical University of Lodz, ul. Muszynskiego 1, 90-151 Lodz, Poland; malgorzata.fabijanska@umed.lodz.pl (M.F.); justyn.ochocki@umed.lodz.pl (J.O.); 2 Department of Theoretical and Structural Chemistry, Faculty of Chemistry, University of Lodz, Pomorska 163/165, 90-236 Lodz, Poland; lilach@uni.lodz.pl; 3 Department of Nucleic Acids Biochemistry, Medical University of Lodz, ul. Pomorska 251, 92-213 Lodz, Poland; leszek.szmigiero@umed.lodz.pl

**Keywords:** palladium(II) complex, 3-hydroxyiminoflavanone, NMR spectroscopy, X-ray crystallography, X-ray wavefunction refinement, cytotoxicity

## Abstract

This work presents the synthesis, spectroscopic properties and single-crystal X-ray examination of the structure of 3-hydroxyiminoflavanone and its palladium complex. It presents the results of NMR (Nuclear Magnetic Resonance) spectroscopy, electron-density studies based on X-ray wavefunction refinement and theoretical calculations combined with QTAIM (Quantum Theory of Atoms in Molecules) and ELI-D (Electron Localizability Indicator) analyses. These offer an interesting new insight into the structures and behavior of flavanone and its complex, in solid state and in solution. The study also examines the cytotoxicity of the ligand and its complex against three human ovarian and lung cancer cell lines.

## 1. Introduction

Over the past 40 years, platinum-based drugs have been widely used in the treatment of many types of tumors including ovarian, testicular, non-small cell lung, head and neck and bladder cancers. Cisplatin (*cis*-[PtCl_2_(NH_3_)_2_], *cis*-diamminedichloridoplatinum) was found to be a particularly effective anticancer drug when administrated as single agent or in combination with other compounds. However, its application is limited by serious side effects such as nephrotoxicity, myelotoxicity, ototoxicity, allergy, and the development of resistance in tumor cells [1,2,3]. Palladium complexes have been studied extensively, based on their structural analogy with platinum complexes. A variety of Pd(II) complexes have shown promising activity as antitumor, antiviral, antimalarial, antifungal and antimicrobial agents [4,5,6]. However, most studies were not very encouraging because the Pd(II) complexes generally showed lower antitumor activity than cisplatin. This could be explained by the more labile nature of palladium complexes compared to their platinum homologues [7]. Nevertheless, some of the palladium complexes revealed higher antitumor activity than cisplatin or carboplatin. The Pd(II) complex of the coumarin-derived ligand demonstrated cytotoxic activity which was almost four orders of magnitude higher than that of carboplatin [8]. Preclinical studies have found flavonoids to have a broad spectrum of desirable effects including anticancer, antioxidant, antiviral, antibacterial and antiallergenic properties. Furthermore, beneficial synergistic interactions have been found between flavonoids and metal ions [9,10]. In addition, complexes with nitrogen-containing ligands are the subject of intensive biological evaluation in the search for less toxic and more selective anticancer agents [11].

Although our present study on 3-hydroxyiminoflavanone (**1**) and its Pd(II) complex (**2**) is a continuation of our preliminary studies on these compounds [12,13], it introduces novel, interesting findings which contrast with those obtained previously. For instance, while 3-hydroxyiminoflavanone was previously claimed to have 3*Z*(syn) geometry in solid state and in solutions [13], our present observations indicate it to have 3*E*(anti) geometry in solid and in polar solvents. It was also found that 3-HIF only has 3*Z*(syn) geometry when dissolved in chloroform, and this exists in equilibrium with the 3*E*(anti) isomer. Additionally, whereas an earlier study proposes that the coordination mode of the palladium complex of 3-HIF was via 4-carbonyl and 3-oxime oxygen atoms [12], our present findings indicate that the ligand chelates the Pd(II) ion via a 4-carbonyl group and a 3-nitrogen atom, while the oxime proton is lost and the ligand eventually becomes 3-nitrosoflavanone in the complex. This new evidence is confirmed by NMR (Nuclear Magnetic Resonance) spectroscopy, single crystal X-ray studies and electron-density studies. This study is the first to compare cytotoxic properties of 3-hydroxyiminoflavonone and its Pd(II) complex with cisplatin towards three cancer cell lines.

## 2. Results and Discussion

### 2.1. Synthesis of the Compounds ***1*** and ***2***

**Scheme 1:**
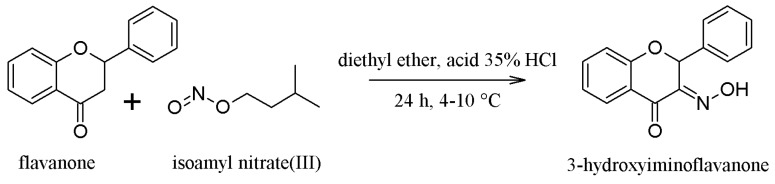
Synthesis of 3-hydroxyiminoflavanone (**1**).

The synthesis of **1** was described elsewhere [13] and reproduced here. In a molecule of flavanone, the carbon atom C3 (adjacent to carbonyl group) is nitrosated with isoamyl nitrate(III) in acidic environment, and the 3-oxime is formed (See Scheme 1). The resulting compound was obtained with good yield and purity (see the Materials and Methods Section), and purified by recrystallization.

**Scheme 2:**
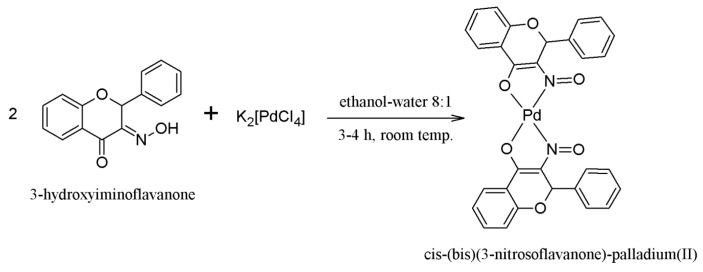
Synthesis of *cis*-(bis)(3-nitrosoflavanone)-palladium(II)—compound **2**.

The synthesis of the palladium complex of 3-hydroxyiminoflavanone (**2**) was also performed earlier (see Scheme 2) [12]. However, whereas previous studies found the chelation to occur via the carbonyl oxygen and oxime oxygen atoms, which would lead to formation of six-membered rings, our results indicate that nitrogen atoms coordinate to the Pd(II) ion, and five-membered rings are formed in the complex. Moreover, the oxime hydrogen atoms are lost and the ligand occurs in the complex as 3-nitrosoflavanone.

### 2.2. Crystallography and Theoretical Calculations

**Figure 1:**
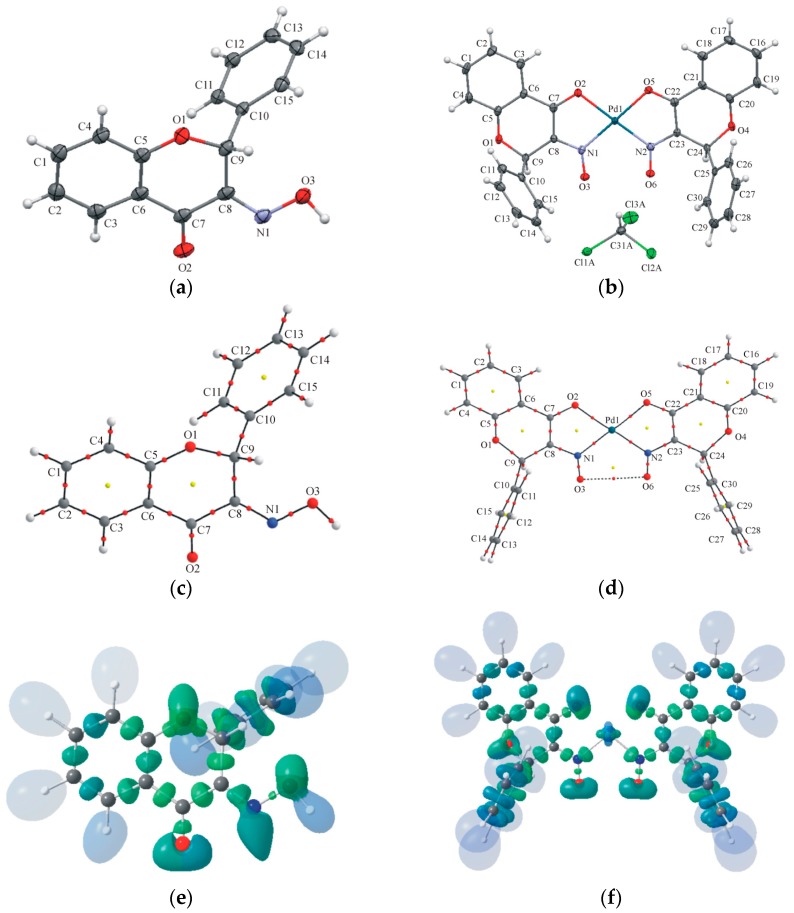
Molecular structures of **1** (**a**) and **2** (**b**) (both IAM models) showing 50% probability ellipsoids and the crystallographic numbering schemes (*MERCURY* [14] representation). Only the major component (*A*) of the disordered solvent molecule is shown. AIM-molecular graphs of XWR (X-ray wavefunction refinement)-model (**1**) (**c**) and OPT-model (**2**) (**d**); bond critical points are given as small red dots, and ring critical points are shown as small yellow dots. Isosurface representation of the localization domains of the ELI-D (an isovalue of Y = 1.40) of XWR-model (**1**) (**e**) and OPT-model (**2**) *(***f**). Transparent mode of the protonated monosynaptic valence basins (hydrogen basins) is applied for clarity reasons.

Figure 1a presents the molecular structure of compound **1** with an atom-numbering scheme, and Table 1 summarizes its crystal data. Molecule **1** consists of a benzene ring (*A*) condensed with a six-membered heterocyclic ring (*C*) that carries a phenyl ring (*B*) and = NOH group. The carbon atom C9 is asymmetric. The system of two condensed rings (*AC*) is rather planar: the maximum deviation of 0.138(1) Å is observed for C9 atom from its least-square plane; the total puckering amplitude calculated for the 10-membered ring is 0.176(2) Å; and the dihedral angle between the best planes of component rings (*A* and *C*) is 4.05(4)°. The phenyl ring (*B*) is oriented almost perpendicular to the system of condensed rings and the corresponding dihedral angle is 87.99(6)°.

**Figure 2:**
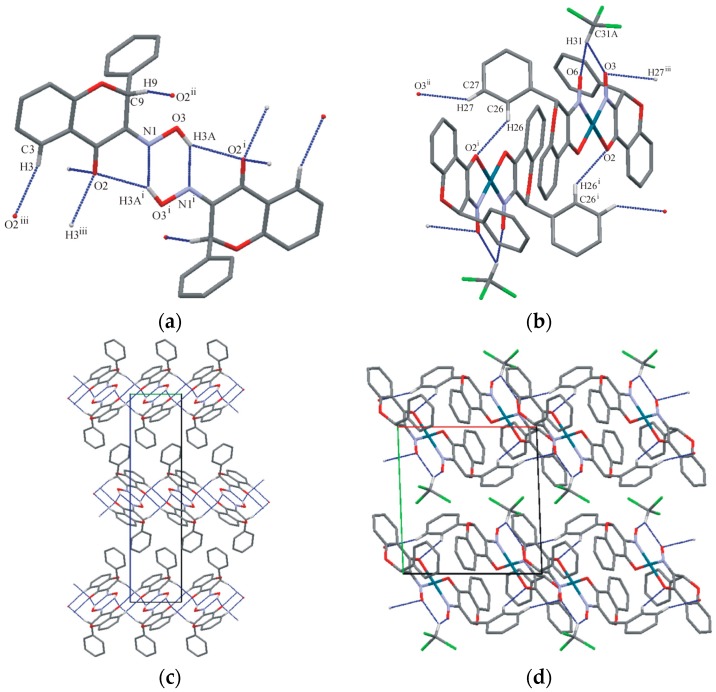
The schemes of intermolecular interactions of free-ligand (**1**) (**a**) and its Pd(II)-complex (**2**) (**b**). Crystal packing of **1** along *a* axis showing the organization of molecular layers *ab* (**c**) and chains of supramolecular dimers in structure **2** viewing along *c* axis (**d**). Symmetry codes: for **1**: (i) 1 − *x*, 1 − *y*, −*z*; (ii) *x*, *y* − 1, *z*; and (iii) −*x*, 2 − *y*, −*z*. Symmetry codes for **2**: (i) 1 − *x*, −*y*, 1 − *z*; (ii) *x* + 1, *y*, *z*; and (iii) *x* − 1, *y*, *z*.

As well as a typical spherical Independent Atom Model (IAM), Hirshfeld Atom Refinement (HAR) [15,16] was used to acquire an aspherical structure model for molecule **1**. Both structural models are in good agreement, the noticeable differences in bond lengths (of 0.013 Å) and valence angles (of 0.6°) only being observed for O3-N1 and O3-N1-C8, respectively. It is important to note that the Hirshfeld-atom model is more accurate than the spherical one, as all geometrical parameters are systematically obtained with smaller standard deviations. The selected bond lengths and valence angles determined for both experimental models of **1** are compared in Table S1 (Supplementary Materials). Figure 2a presents a scheme of intermolecular interactions characteristic of compound **1**, and the geometries of interactions are given in Table 2. Two intermolecular hydrogen bonds, O3-H3A···O2^i^ and O3-H3A···N1^i^ (symmetry code (i): 1 − *x*, 1 − *y*, −*z*) are responsible for the formation of a cyclic dimer about a center of symmetry. As a result, a contact N1···N1 of 2.727(2) Å is also observed between the interacting molecules, which is shorter than the sum of the corresponding van der Waals radii (3.10 Å). Furthermore, the supramolecular structure of **1** is dominated by weak C-H···O interactions: C9-H9···O2(*x*, *y* − 1, *z*) and C3-H3···O2(−*x*, 2 − *y*, −*z*), which link the adjacent dimers into two-dimensional molecular layers in the *ab* plane. The layer-structure is additionally stabilized by weak C1-H1···π (ring *C*; *x* − 1, *y*, *z*) interactions and close C···O contacts: C1···O3(*x* − 1, *y*, *z*) (3.204(2) Å) and O1···C3(*x*, *y* − 1, *z*) (3.206(2) Å). No specific interactions between adjacent layers were found. The X-ray diffraction study of compound **2** shows that the asymmetric unit contains a palladium cation and two flavanone ligands in anionic form arranged in a *cis*-configuration; additionally, a trichloromethane solvent molecule is also observed. The perspective view of molecular structure of **2** is shown in Figure 1b. The Pd(II) center is four coordinated in *bis*-bidendate fashion via two N(oxime) and two O(carbonyl) atoms, thus the coordination geometry is distorted square planar with angles varying from 81.25(6)° to 99.92(6)° (Table S1 in Supplementary Materials).

The Pd-N [1.9898(16) Å and 1.9809(16) Å] and Pd-O [2.0586(13) Å and 2.0636(13) Å] bond lengths are comparable with those reported for related Pd(II) complexes [17,18]. The coordination of the Pd(II) ion results in the formation of two five membered chelate rings, Pd1/O2/C7/C8/N1 and Pd1/O5/C22/C23/N2, which are essentially planar and form a dihedral angle of 3.21(7)°.

The Cremer and Pople’s ring puckering parameters [19] calculated for the six-membered heterocyclic rings: O1/C5-C9 [*Q_TOT_* = 0.2651(19) Å, θ = 119.6(4)°, ϕ = 140.8(5)°] and O4/C20-C24 [*Q_TOT_* = 0.2190(18) Å, θ = 65.7(5)°, ϕ = 326.1(6)°] show that both adopt skew-boat conformation in contrast to free ligand **1**, where the corresponding ring was essentially planar. Accordingly, the asymmetry parameters [20] [ΔC_2_(O1-C9)=3.9(3)° and ΔC_2_(O4-C24) = 1.2(3)°] imply the existence of the pseudo-twofold-axis crossing the mid-points of the opposite bonds of the analyzed rings. The planar fragments of the phenyl ring and benzene ring molecules (condensed with a puckered six-membered ring) form angles of 79.19(9)° and 77.8(1)°, respectively. In describing complex structure **2,** it is important to note that upon coordination, flavanone ligand **1** undergoes significant evolution from its oxime to nitroso form (additionally anionic), demonstrated by its experimentally and theoretically obtained geometrical, topological and integrated parameters.

The topological analysis of the investigated flavanone ligand **1** and Pd-complex **2** structures was performed based on the Quantum Theory of Atoms in Molecules (QTAIM) [21]. While two models of electron-density distribution were considered for compound **1**, *i.e.*, an experimental model obtained from X-ray wavefunction refinement (**1**-XWR) [22] and a theoretical one derived by DFT-geometry optimization (**1**-OPT), only a theoretical electron-density model was available for compound **2**. The molecular graphs of XWR-model of **1** and OPT-model of **2** are presented in Figure 1c,d and compared with corresponding ELI-D-representations (Figure 1e–f). The combination of the QTAIM and ELI-D [23,24] approaches provides quantitative information concerning the nature of the bonding present in the analyzed structures. Table 3 presents the topological properties of the bonds derived from QTAIM space partitioning and a set of ELI-D derived properties from the models of **1** and **2**.

Overall, since systematically slightly longer distances in DFT-models are expected and acceptable, the agreement between the experimentally-observed and theoretically-calculated structures is very good and structural trends are maintained.

The analysis of geometrical parameters reveals a considerable shortening of bond lengths C7-C8/C22-C23 and O3-N1/O6-N2 (ligand **1**/ligand **2**) in complex structure **2** in comparison to C7-C8 and O3-N1 in the free ligand structure of **1**. Concomitantly, a significant lengthening of bond distances O2-C7/O5-C22 and N1-C8/N2-C23 is observed.

Such geometrical changes are reflected in topological parameters determined in bond critical points (bcps); electron density values at bcp increased or decreased when the distance was shortened or lengthened, respectively. Furthermore, these trends are confirmed by the bond ellipticity value (ε) [25] and delocalization index (δ) [26,27]. Literature ellipticity equals 0 for a perfectly isolated single C-C bond and ε = 0.23 for a 1.5-fold bond in benzene; a delocalization index value of δ_A,B_ = 1 is assigned to a single Lewis pair equally shared between two atoms, A and B, while a value higher than 1 is awarded when the two atoms share more than one electron pair between their respective basins.

In our study, the following parameters amount ε_C7-C8_ = 0.15 and δ_C7,C8_ = 0.95 in were found **1** (the XWR model) and both increase upon complexation to 0.22 and 1.14, respectively (**2**-OPT model). On the other hand, considerable greater δ values are encountered for the formal double bonds O2-C7 and N1-C8 in **1** (δ_O2,C7_ = 1.37, δ_N1,C8_ = 1.50) than in **2** (δ_O2,C7_ = 1.19, δ_N1,C8_ = 1.22). A reversed trend is observed for the N1-O3 bond, δ_N1,O3_ = 1.34 in **2** *vs.* δ_N1,O3_ = 1.66 in **1**, which is additionally supported by increasing the value of the ratio *H*/ρ_bcp_[28], indicating a degree of bond covalence.

**Figure 3:**
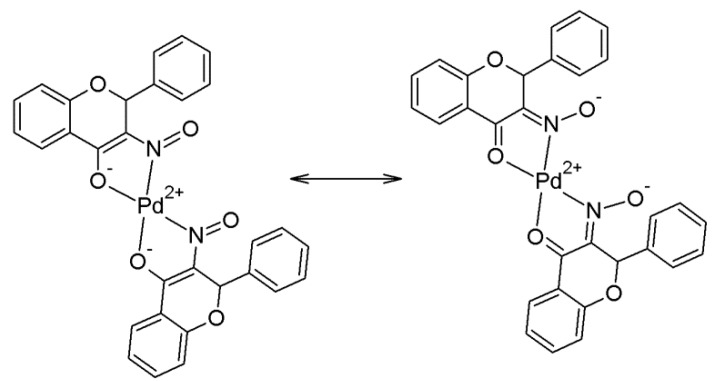
Two resonance forms of Pd(II) complex **2**.

To summarize, in Pd(II) complex structure **2**, the structural fragments within the flavanone moieties O2-C7-C8-N1-O3 (ligand **1**) and O5-C22-C23-N2-O6 (ligand **2**) clearly contain delocalized π-systems. Figure 3 presents two possible resonance forms of **2**. In the solid state, form 1 (Figure 3) of complex structure **2** is determined. Table S2 summarizes the population of (non-bonding) lone pair basins obtained using the ELI-D technique. Two basins belonging to the lone pair region of the oxygen O2 (carbonyl) atom in structure **1** can be distinguished, each around 8 Å^3^ populating 5.24 *e* in total, whereas this space is contracted in complex **2**, the population being reduced to 3.56 *e* (O5 atom in ligand 2). In contrast, the population of lone pairs of oxygen O3(oxime) atoms increases from 4.68 *e* integrated in free neutral ligand **1** to 5.62 *e* in structure.

Moreover, an inspection of Figure 1e–f, Table 3 and Table S4 (Supplementary Materials) clearly indicates that the oxygen O2 and O5 atoms form a fairly strong ionic interaction to the metal center Pd(II), as confirmed by a Raub-Jansen index [29,30] of 97% and the highest value of degree of iconicity (*G*/ρ_bcp_) observed in structure **2**.

Pd1-N1 and Pd1-N2 bonds have more of a polar character (*RJI* = 94.5%) with relatively greater electron density in bcp and smaller *G*/ρ_bcp_ ratio, but higher *H*/ρ_bcp_ and δ_N1,Pd1_ in comparison to O-Pd bonding.

The crystal packing of **2** is dominantly arranged by weak interactions. First of all, the complex molecule interacts with the solvent molecule via two C31A-H31···O3 and C31A-H31···O6 interactions (Table 2). Such molecular pairs form supramolecular centrosymmetric dimers with C26-H26···O2 (1 − *x*, −*y*, 1 − *z*) interaction (Figure 2b), which subsequently organize into infinite chains along the crystallographic *a* axis by C27-H27···O3(*x +* 1, *y*, *z*) contacts. The final packing arrangement of **2** is shown in Figure 2d. Finally, it is interesting to note that the QTAIM analysis of close intramolecular O3···O6 contact (2.878 Å—IAM model, 2.977 Å—OPT model) shorter than the sum of van der Waals radii of interacting atoms (3.04 Å) revealed the following topological parameters at its bcp: electron density ρ_bcp_ = 0.06 *e*Å^−3^ and associated Laplacian ∇^2^ρ_bcp_ = 0.7 *e*Å^−5^ (Figure 2d). This weak non-covalent interaction seems not to affect the ELI-D lone-pair basins of O3 and O6 oxygen atoms (Figure 1f), suggesting that it results from the spatial arrangement of coordinating flavanone ligands.

### 2.3. NMR Spectroscopic Analysis and Solution Stability Studies

#### 2.3.1. Compound **1**

**Figure 4:**
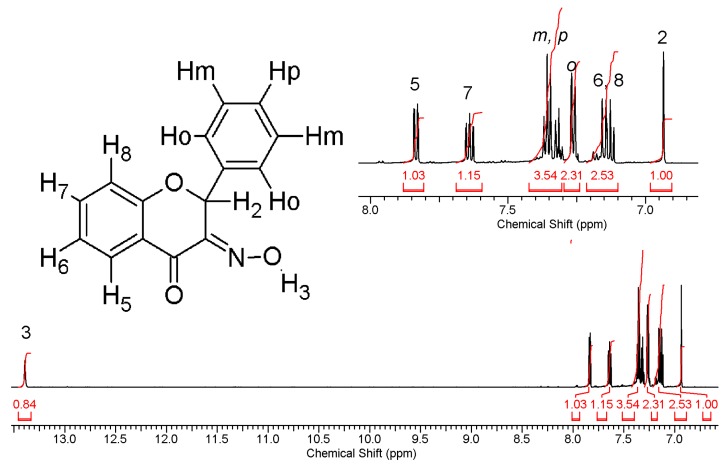
^1^H-NMR (Nuclear Magnetic Resonace) spectrum of **1** in DMSO-*d*_6_, with integration values and assignments.

The results of the ^1^H-NMR analysis of **1** in DMSO-*d*_6_ are generally in agreement with those described by Kostka and Zyner [13]. The signals between 6.9–7.9 ppm are typical for the flavanone moiety. The oxime proton signal is visible at 13.39 ppm, and its integration value suggests that the molecule only has an oxime tautomeric form (Figure 4). However, our results differ regarding the significance of the observed downfield shift of the oxime proton signal: while Kostka and Zyner [13] suggest that the shift indicates 3*Z*(syn) geometry of the molecule, our results indicate that the ligand exists as an *E* (anti) isomer rather than *Z* (syn), both in solid form and in DMSO solution. The downfield-shifted signal of the oxime proton is not necessarily due to the presence of an intramolecular hydrogen bond in 3*Z*(syn)-HIF [13], but it is typical for the oxime proton in similar molecules [31,32,33]. Additionally, the signals of aromatic protons from the phenyl ring B of the flavanone moiety are split in two parts (7.24–7.28 ppm for the 2H bound to the *ortho* carbon atoms, and 7.30–7.38 ppm for the 3H bound to the *meta* and *para* carbon atoms, ring C), which indicates the presence of steric interaction between the phenyl group and the oxime –OH group in the anti position. Therefore, the ^1^H-NMR spectrum of **1** in DMSO-*d*_6_ displays the pattern for 3*E*-hydroxyiminoflavanone (Figure 4). However, when the ^1^H-NMR spectrum of **1** is made using CDCl_3_ as solvent, the pattern is different (Figure S2 in Supplementary Materials). The signal of the labile proton is very weak and very broad (often hardly visible), at 10.15 ppm (Figure S3 in Supplementary Materials). The signals of aromatic protons from the phenyl ring B of the flavanone moiety are not split, but are seen as a large multiplet, suggesting that the aromatic protons of ring B do not interact with the polar oxime group. Even so, after 24 h, a small singlet exchangeable with D_2_O appears at 14.91 ppm (Figure S2), which could possibly be the signal from the –NOH group, along with additional signals at 6.29, 7.12, 7.17, 7.65 and 7.95 ppm, which indicates the formation of an equilibrium between two isomeric forms of **1**.

After six days in solution, the emerging isomer constitutes almost 30% of the total amount, and its signal pattern is similar to that seen in DMSO-*d*_6_, indicating the formation of 3*E*-HIF (Figure S2 in Supplementary Materials). That suggests that the initial form **1** takes after dissolution in chloroform is 3*Z*-HIF. This has been confirmed by Enchev *et al.* [33] in the case of 4-nitroso-5-pyrazolones, and by Belmar *et al.* [32] in the case of 1-(2-hydroxyethyl)-3-methyl-4-hydroxyimino-5-pyrazolone, where a polar solvent (DMSO) supports the formation of the *E* diastereoisomer, and a non-polar solvent, *i.e.*, chloroform, supports the formation of the *Z* form. Enchev *et al.* [34] describe the tautomeric and conformational equilibrium of 9,10-phenantrenequinonemonooxime, where the most prevalent form of the molecule in chloroform is the *Z* isomer, and in DMSO the *E* isomer. The nitroso tautomers are not thermodynamically preferred, and unlikely to exist in solution [33]. In the case of 3-HIF, the existence of the nitroso-enol tautomer is not supported by the data. ^13^C-NMR spectra recorded in DMSO-*d*_6_ and CDCl_3_ reveal a signal assigned to the carbonyl carbon atom near 177 ppm (Figure S5 in Supplementary Materials), which is typical for carbonyl carbon atoms C=O [31,32]. Signals from C-OH carbon atoms should be right-shifted towards around 160 ppm [32,35].

#### 2.3.2. Compound **2**

**Figure 5:**
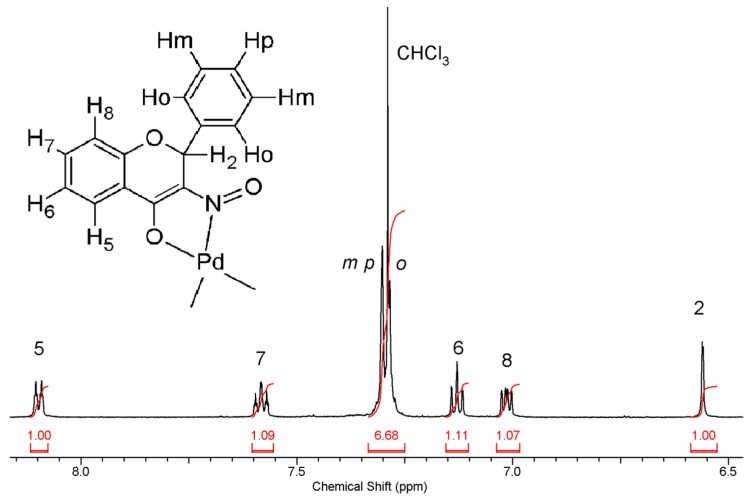
^1^H-NMR spectrum of **2** in CDCl_3_, with integration values and assignments.

Again, our results are in contrast with those of previous preliminary studies, which suggest that only oxygen atoms coordinate with Pd(II) [12]. This claim was made on the basis of IR spectra, which only indicated the presence of Pd-O bonds. However, such an interpretation can be misleading, due to overlapping signals of similar, but not identical types of bonds. Our crystallographic data show that coordination bonds are formed by nitrogen and oxygen atoms, and the oxime hydrogen atoms are lost during the coordination. This is also in agreement with ^1^H-NMR data, where no signal from labile proton is observed (Figure 5).

As the complex was used in biological *in vitro* study, its stability was determined using UV-Vis spectroscopy, in an aqueous environment at 37 °C to mimic the conditions in the cell culture incubator. The absorption spectrum has two maxima, at 316 nm and at 461 nm. Compound **2** in solution seems to be stable, and the spectrum shows practically no changes for up to 62 h of incubation, with no shifts of the maxima. The spectra are shown in the Figure S4 in the Supplementary Materials.

### 2.4. Cytotoxicity Assay (3-(4,5-Dimethylthiazol-2-yl)-2,5-diphenyltetrazolium Bromide or MTT Assay)

The three cell lines used, A2780, A2780cis and A549, have different degrees of sensitivity to cisplatin. The most sensitive line is A2780 (human ovarian carcinoma), with IC_50_ below 1 µM. A2780cis is a commercially-available subline resistant to cisplatin, with an IC_50_ about 20 µM. The A549 cells (human non-small cell lung carcinoma) have relatively high intrinsic resistance to cisplatin, with an IC_50_ of approximately 4 µM (for details see Table 4, and Figure S6 in Supplementary Materials). Compound **1** is less cytotoxic than **2** and CDDP, with only a small antiproliferative effect up to a concentration of 25 µM (See Figure S6). IC_50_ values are given in Table 4. However, the fact that **1** is significantly cytotoxic at the concentration below 100 µM suggests that the choice of ligand substantially contributes to the toxicity of the complex **2**. As compound **2**, *cis*-(bis)(3-nitrosoflavanone) palladium(II), was insoluble in water and barely soluble in DMSO, the maximal concentration tested in the cell culture was 15 µM. The low solubility of **2** is possibly due to the lack of polar moieties on the surface of the molecule, and its electric neutrality. It was not possible to determine the IC_50_, because at this concentration the decrease in the cell proliferation was less than 50%. The result confirms the general observation, that Pd(II) compounds are less toxic than Pt(II) compounds [7]. Therefore, palladium compounds are unlikely to be an alternative for cisplatin as cytostatic agents, unless they contain very active coordinated ligands. More studies may be needed, to account for the many factors affecting the cytostatic properties of metal complexes. For example, although for a long time, *trans* complexes of Pt(II) were believed to be inactive, many were later proven to be cytostatic [36,37]. There is also a possibility that a compound, while not being cytotoxic, may possess anticancer activity. For example, imidazolium *trans*-DMSO-imidazole-tetrachlororuthenate(III) (NAMI-A, for New Anti-tumor Metastasis Inhibitor) is not directly cytotoxic for most solid cancers, but acts as an anti-metastatic agent [38]. Moreover, while widely believed to be not toxic for tumors, NAMI-A was found to be highly cytotoxic for certain leukemia cell lines [39].

## 3. Materials and Methods

### 3.1. Synthetic Procedures

#### 3.1.1. Synthesis of Compound **1**—(3-Hydroxyminoflavanone or 3-HIF)

The synthesis was based on the procedure described by Kostka and Zyner [13], with small modifications. Briefly, 6 g of flavanone (Alfa Aesar, Karlsruhe, Germany) was dissolved in 450 mL of diethyl ether. Then, 9 mL of isopentyl nitrate(III) and 15 mL of 35% hydrochloric acid were added. The mixture was gently shaken and left at 4–10 °C for 24 h. The next day, the acidic layer was separated and discarded, and the ether solution was vigorously shaken with 150 mL of 1.5%–2% aqueous NaOH solution to extract the product as the sodium salt. The extraction was repeated twice. The alkalized water fraction was collected, cooled in an ice-water bath and acidified with pure acetic acid to pH ≈ 3. The resulting yellow precipitate of 1 was filtered off, washed with water and air dried. The crude product was recrystallized from hot toluene–methanol (10:1) solution and air dried again. M.w. = 253 g/mol; El. anal. Measured (calc.%): C—71.24 (71.14), H—4.10 (4.38), N—4.42 (5.53); M.p. 184–185 °C, ^1^H-NMR (600 MHz, DMSO-*d*_6_), δ ppm 6.93 (s, 1H) 7.10–7.17 (m, 2 H) 7.24–7.28 (m, 2H) 7.30–7.38 (m, 3H) 7.64 (ddd, *J* = 8.56, 7.06, 1.69 Hz, 1H) 7.83 (dd, *J* = 7.91, 1.88 Hz, 1H) 13.35–13.44 (m, 1H). A single crystal for X-ray analysis was obtained by slow evaporation of ethyl acetate from the solution of 1 at room temperature.

#### 3.1.2. Synthesis of Compound **2**—[*cis*-(bis)(3-Nitrosoflavanone)-palladium(II)]

The synthesis was based on the procedure described in [12] with modifications. Briefly, 2 mmol (506 mg) of yellow powder **1** was dissolved in 80 mL 96% ethanol and stirred. Following this, 1 mmol (326 mg) of potassium tetrachloropalladate(II) was dissolved in 10 mL of distilled water, added slowly to the ethanolic solution of **1** and left stirring at ambient temperature for 3–4 h. Then, the orange precipitate was filtered off, and the solution was diluted with distilled water to precipitate more of the product. It was filtered off and the whole batch of the crude product was washed with water and air dried. The dry product was dissolved in chloroform, filtered and left for evaporation to obtain a red crystalline precipitate; then ground with diethyl ether and air dried again to remove any chloroform residues. M.w. = 611 g/mol; El. anal. Measured (calc.%): C—58.41 (58.98), H—3.25 (3.30), N—4.54 (4.59); M.p. 211–213 °C, IR (KBr disc) in cm^−1^: 1608s, 1577s, 1528s, 1487s, 1467 s, 1441s, 1413s, 1325m, 1280m, 1261m, 1213m, 1199 m, 1148 s, 1108 m, 1078 w, 1022 m, 960 w, 916 w, 862 m, 845w, 769m, 751s, 724m, 796m, 667m, 627w, 595w, 557w, 518w, 468w. (s—strong, less than 25% transmittance; m—medium, 25%–55% transmittance; w—weak, 55%–70% transmittance). ^1^H-NMR (600 MHz, CDCl_3_), δ ppm: 6.56 (d, *J* = 1.13 Hz, 2H) 7.01 (dd, *J* = 8.47, 5.46 Hz, 2 H) 7.10–7.16 (m, 2H) 7.25–7.35 (m, 13H) 7.54–7.62 (m, 2H) 8.10 (dt, *J* = 7.91, 1.69 Hz, 2H). A single crystal for X-ray analysis was obtained by slow evaporation of chloroform from the solution of 2 at room temperature.

### 3.2. Crystallography and Theoretical Calculations

#### 3.2.1. X-ray Measurements and Spherical Structure-Refinements (IAM-Independent-Atom Models)

The crystal and molecular structures of 1–2 were determined by single-crystal X-ray diffraction. Data collection was carried out at 100K with an Agilent SuperNova diffractometer with an Atlas detector (Agilent Technologies, Yarnton, Oxfordshire, UK) (1) and an Oxford Xcalibur diffractometer with a Sapphire3 detector (Oxford Diffraction, Abingdon, UK) (2), both using Mo*K*_α_ radiation and ω scan. The data reduction and multi-scan absorption correction were performed by CrysAlis PRO [40] and CrysAlis RED [41] software for **1** and **2**, respectively. Both structures were solved by direct methods and refined by full-matrix least-square procedures on *F*^2^ using the SHELXL-2013 [42] program package implemented in WinGX [43]. All non-hydrogen atoms were refined anisotropically. The (O)-H-atom (H3A) involved in the O-H···O/N hydrogen bonds in structure **1** was located in a Fourier map and refined freely. The remaining (C)-H atoms were calculated to their idealized positions and refined as riding atoms with isotropic displacement parameters *U*_iso_(H) = 1.2*U*_eq_(C). Three chlorine atoms (Cl1, Cl2, Cl3) and one carbon atom (C31) in the solvent molecule (trichloromethane) of structure **2** were found to be disordered and refined in two alternative positions with the final site-occupation factors: *k*_A_:*k*_B_ = 0.5889(16):0.4111(16). The solvent-hydrogen atom (H31) was refined freely. The crystallographic data and the final figures of merit for spherical refinements of **1**–**2** are presented in Table 1.

#### 3.2.2. X-ray Wavefunction Refinement (XWR)

The geometry of ligand molecule 1 obtained from spherical refinement (IAM model, all H-atoms refined freely) was used as input for the Hirshfeld-atom refinement [15,16] (HAR). In HAR, the following structural parameters were refined freely: coordinates for all atoms, anisotropic displacements parameters (ADPs) for non-hydrogen atoms and isotropic displacement parameters (*U*_iso_) for H-atoms. The final geometry of **1**, obtained by Hirshfeld-atom refinement was used as input for X-ray constrained wavefunction fitting (XCW). In the fitting procedure, the obtained wavefunction was constrained to the experimental data by introducing the Lagrange parameter; final λ = 0.10. Both steps of X-ray wavefunction refinement [22] (XWR = HAR + XCW) were performed using blyp/cc-pVTZ [44,45,46] level of theory within the Tonto [47] software package. In order to simulate the effect of the crystal environment, a cluster of charges and dipoles around the central molecule was introduced: all molecules with at least one atom within a radius of 8 Å around the central molecule were included in the surrounding cluster. All calculations were carried out on *F*. Negative intensities |*F*|^2^ as well as reflections with |*F*| < 2.0σ(|*F*|) were pruned from the data. Hence the number of data used in spherical refinement and XWR differs. The final figures of merit are summarized in Table 1. The residual density map (Figure S1a) is essentially featureless, and the deformation density plot (Figure S1b) shows only the expected characteristics.

#### 3.2.3. Theoretical Calculations

Starting from the experimental coordinates (taken from IAM models) gas-phase structures of **1** and **2** were obtained by full geometry optimizations at the same level of theory, blyp/cc-pVTZ, as X-ray wavefunction refinement. In the case of **2**, the effective core potential for the Pd atom (ECP28MDF) [48] was used along with the associated triple-ζ basis set, the cc-pVTZ basis set was also used for all other atoms. The final geometries were confirmed to be minima by an analysis of harmonic vibrational frequencies. All computations were performed using the Gaussian09 [49] program.

#### 3.2.4. QTAIM (Quantum Theory of Atoms in Molecules) and ELI-D (Electron Localizability Indicator) Analyses

QTAIM [21] and ELI-D [23,24] analyses. The wavefunction derived by the XCW procedure and those obtained from DFT-optimizations were analyzed using AIMALL [50] to calculate all the bond and atomic properties presented in this study. Moreover, the X-ray constrained wavefunction of **1** and theoretically obtained checkpoint files of **1** and **2** (OPT models) were used in ELI-D analysis. For the grid calculations with DGRID-4.6 [51], a step size of 0.05 bohr was applied. The AIM graphs were displayed with AIMAll (Figure 1c,d). ELI-D graphs were created with MOLISO [52] (Figure 1e,f).

### 3.3. NMR Spectroscopic Analysis

NMR spectra were collected at the Laboratory of Molecular Spectroscopy, Faculty of Chemistry, University of Lodz, Poland, on a Bruker Avance III 600 MHz spectrophotometer (Bruker Corporation, Billerica, MA, USA), at room temperature, in DMSO-*d*_6_ or CDCl_3_. The ^13^C-NMR spectrum of 1 in DMSO-*d*_6_ was recorded on apparatus Bruker (Bruker Corporation) 300 MHz at the Medical University of Lodz.

### 3.4. Solution Stability Studies: UV-Vis Spectroscopy

UV-Vis spectra were collected using Spectrostar Nano spectrophotometer (BMG LABTECH GmbH, Ortenberg, Germany). The complex **2** was dissolved in DMSO at the concentration 7 mM, and then diluted in water at 15 µM, to achieve DMSO concentration 0.2% *v*/*v*. The solution was incubated at 37 °C in a covered quartz cuvette, and the spectra were collected during 62 h of incubation.

### 3.5. Cell Culture and Cytotoxicity Assay

#### 3.5.1. Cell Culture

The cell lines A2780 (human ovarian carcinoma, ECACC cat. No. 93112519), A2780cis (subline resistant to cisplatin, ECACC cat. No. 93112517) and A549 (human non-small cell lung carcinoma, ECACC cat. No. 86012804) were purchased from Sigma-Aldrich. The cells were cultured in RPMI-1640 medium (Biological Industries, Kibbutz Beit Haemek, Israel) supplemented with 10% heat inactivated fetal bovine serum, 5 mM Hepes and 50 µg/mL gentamycin. A549 cells were cultured in high-glucose DMEM medium (Biological Industries) supplemented with 10% heat inactivated fontal bovine serum, 5 mM Hepes and 50 µg/mL gentamycin. All cell lines were maintained in a humidified incubator at 37 °C, 5% CO_2_. The cell lines were grown as monolayers, and renewed every 3–4 days.

#### 3.5.2. Cytotoxicity Assay (3-(4,5-Dimethylthiazol-2-yl)-2,5-diphenyltetrazolium Bromide or MTT Assay)

The cells were seeded in 24-well plates (TPP), 1 mL of cell suspension per well, at the following densities: A2780—10^4^ cells/mL, and A2780cis and A549—1.5·10^4^ cells/mL. The next day, the tested compounds (**1**, **2** and CDDP) were added, pre-dissolved in DMSO (at the max. concentration 7.5 mM) and then dissolved in the complete culture medium, at concentrations twice those of the final ones, and 1 mL of resulting solution was added per well. The solutions of **2** and CDDP compounds were freshly made before every experiment to avoid the solvolysis [53,54]. Stock solution of **1** was stored refrigerated. Final concentration of DMSO in culture medium was 0.2% *v*/*v*. After 72 h, MTT solution in PBS was added to a final concentration of 0.25 mg/mL in culture. The suspension was then incubated at 37 °C until visible color appeared (approximately 1 h). The culture medium was then removed, and the purple formazan crystals dissolved in 1 mL of DMSO. The absorbance was read at λ = 540 nm on a Spectrostar Nano microplate reader. Every concentration was measured in triplicate, and each experiment was repeated on at least three separate plates. The results are displayed as percentages of the solvent control (assumed to be 100%), and shown as the mean value ± standard deviation (see Figure S6 in Supplementary Materials).

## 4. Conclusions

The work presents a detailed description of the structure of 3-hydroxyiminoflavanone and its palladium(II) complex, and sheds new light on their structures in solution and in solid state. It shows that 3*E*(anti)-hydroxyiminoflavanone (**1**) reacts with tertrachloridopalladate(II) and forms complex **2**, while the ligands lose their oxime proton. The polar environment of the reaction may affect the structure of the ligand, and therefore the complex, by promoting the existence of the *E* (anti) form of **1**. Non-polar solvents, such as chloroform, probably support the formation of *Z* (syn) isomer of **1**. A combination of X-ray single-crystal structure determination and electron-density studies of **2** show that *cis*-(bis)(3-nitrosoflavanone)-palladium(II) is formed, and the donor atoms of the ligand are the carbonyl oxygen and oxime nitrogen. 3-hydroxyiminoflavanone thus becomes 3-nitrosoflavanone when coordinated to the Pd(II) ion. An analysis of the real-space bonding indicators considered in this study, *i.e.*, all geometrical, topological and integrated parameters describing properties of chemical bonds, reveals π-delocalization within the flavanone moieties of structure **2**. Probably due to the lack of any polar moieties on the outer surface of the molecule, the emerging complex is hardly soluble in polar solvents. At a concentration of 15 µM in the culture media, compounds **1** and **2** are only moderately cytotoxic towards three human cancer cell lines, and the little difference observed in cytotoxicity towards cisplatin-sensitive and cisplatin-resistant sublines of A2780 suggests that they do not distinguish between the two cell lines.

## Figures and Tables

**Table 1:** Crystallographic data of **1** and **2.**

	1	2
Empirical formula	C_15_H_11_NO_3_	C_30_H_20_N_2_O_6_Pd·CHCl_3_
Formula weight	253.25	730.25
Crystal system, space group	Monoclinic, *P*2_1_/n	Triclinic, *P*$\bar{1}$
*a*, *b*, *c* (Å)	8.6182(17), 5.7715(12), 23.468(5)	10.3894(7), 10.9265(8), 13.1736(8)
α, β, γ (°)	90.00, 94.06(3), 90.00	83.848(6), 80.996(5), 86.933(6)
*V* (Å^3^)	1164.4(4)	1467.50(17)
*Z*	4	2
*D*_x_ (Mg/m^3^)	1.445	1.653
Crystal size (mm)	0.25 × 0.06 × 0.03	0.24 × 0.13 × 0.07
Crystal habit and color	Plate, colorless	Plate, red
λ (Å)	0.71073 (Mo*K*_α_)	0.71073 (Mo*K*_α_)
μ (mm^−1^)	0.10	0.95
*T* (K)	100(2)	100(2)
Measured/unique reflections	18731/3055	12395/6905
*R*_int_	0.021	0.023
θ range (°)	2.5–29.0	3.1–28.0
Completeness to θ_max_ (%)	98.9	97.2
IAM model (spherical)		
Observed reflections [*I* > 2σ(*I*)]	2741	5695
Data/restraints/parameters	3055/0/176	6905/0/429
Refinement method	Full-matrix least-squares on *F*^2^	Full-matrix least-squares on *F*^2^
*S*	1.112	0.927
*R* [*I* > 2σ(*I*)], *wR* [*I* > 2σ(*I*)]	0.0452, 0.1009	0.0257, 0.0517
*R* (all data), *wR* (all data)	0.0497, 0.1034	0.0351, 0.0527
(Δ/σ)_max_	<0.001	0.001
Δ*ρ*_max_ (*e*Å^−3^), Δρ_min_ (*e*Å^−3^)	0.40, −0.19	0.50, −0.75
XWR: HAR model (aspherical)		
Refinement method	on *F* with weight = 1/σ(|*F*|)	
Functional/basis-set	blyp/cc-pVTZ	
Data/restraints/parameters	2974/0/216	
χ^2^	10.17	
*S*	3.19	
*R* (*F*), *wR* (*F*)	0.0340, 0.0233	
XWR: XCW (fitting procedure)		
Refinement method	on *F* with weight = 1/σ(|*F*|)	
Functional/basis-set	blyp/cc-pVTZ	
Data/restraints/parameters	2974/0/1	
χ^2^ (λ = 0.10)	6.42	
*S*	2.53	
*R* (*F*), *wR* (*F*)	0.0303, 0.0192	

**Table 2:** The geometries of intermolecular interactions of **1** and **2** ^a^.

Interaction	D-H	H···A	D···A	D-H···A
**1**				
O3-H3A···O2 ^i^	0.96(2)	2.49(2)	3.3359(18)	146(2)
O3-H3A···N1 ^i^	0.96(2)	1.98(2)	2.7996(18)	142(2)
C9-H9···O2 ^ii^	0.98	2.41	3.283(2)	147
C3-H3···O2 ^iii^	0.93	2.46	3.297(2)	149
C1-H1···*Cg* ^ii,b^	0.93	2.79	3.628(2)	150
**2**				
C31-H31···O3	0.99(2)	2.26(2)	2.990(4)	130(2)
C31-H31···O6	0.99(2)	1.29(3)	2.964(4)	124(2)
C26-H26···O2 ^i^	0.93	2.51	3.259(2)	137
C27-H27···O3 ^ii^	0.93	2.63	3.395(2)	140

^a^ distances D-H, H···A and D···A in Å, angle D-H···A in deg; ^b^ Cg—is the centroid of the phenyl ring C (C10-C15); Symmetry codes: for **1** ^i^ 1 − *x*, 1 − *y*, −*z*; ^ii^ *x*, *y* − 1, *z* ; ^iii^ −*x*, 2 − *y*, −*z*; for **2** ^i^ 1 − *x*, −*y*, 1 − *z*; ^ii^ *x* + 1, *y*, *z*.

**Table 3:** Geometrical ^a^, topological ^b^ and integrated ^c^ bond descriptors determined for selected bonds in XWR-model of **1** and OPT-model of **2** ^d^.

Bond	*d*	ρ_bcp_	∇^2^ρ_bcp_	ε	*G*/ρ_bcp_	*H*/ρ_bcp_	δ	*V*_ELI_	ELI_pop_	Y_max_	Δ_ELI_	*RJI*
**(1-XWR)**												
O1-C5	1.3520(6)	1.94	−7.6	0.04	1.15	−1.43	0.96	1.16	1.39	1.56	0.06	78.4
O1-C9	1.4405(6)	1.61	−9.6	0.07	0.87	−1.29	0.80	1.21	1.40	1.58	0.06	82.3
O2-C7	1.2218(5)	2.75	−10.9	0.16	1.42	−1.70	1.37	4.28	2.29	1.54	0.04	73.2
O3-N1	1.3641(5)	2.24	−7.6	0.03	0.67	−0.90	1.34	0.56	0.99	1.46	0.02	55.0
N1-C8	1.2856(6)	2.57	−22.8	0.39	0.92	−1.54	1.50	6.88	3.08	1.71	0.17	69.2
C5-C6	1.4026(6)	2.09	−23.3	0.25	0.36	−1.14	1.25	7.92	2.87	1.82	0.03	54.2
C6-C7	1.4614(7)	1.96	−21.7	0.12	0.27	−1.05	1.04	4.84	2.38	1.95	0.01	50.8
C7-C8	1.4839(7)	1.89	−20.2	0.15	0.26	−1.01	0.95	5.22	2.40	1.97	0.03	52.1
C8-C9	1.5015(7)	1.79	−17.9	0.15	0.26	−0.96	0.94	3.82	2.21	1.95	0.05	55.9
**(2-OPT)**												
N1-Pd1	2.032	0.80	8.2	0.07	1.07	−0.36	0.76	6.42	2.53	1.77	0.09	94.5
O2-Pd1	2.112	0.57	8.6	0.04	1.27	−0.22	0.56	2.80	2.02	1.61	0.03	97.1
O1-C5	1.364	1.97	−14.1	0.05	0.95	−1.45	0.97	1.23	1.47	1.59	0.05	76.1
O1-C9	1.485	1.51	−11.3	0.03	0.61	−1.14	0.83	1.10	1.24	1.56	0.06	76.4
O2-C7	1.286	2.40	−15.5	0.07	1.14	−1.60	1.19	1.94	1.82	1.57	0.01	73.7
N1-O3	1.249	3.15	−21.7	0.06	0.74	−1.22	1.66	0.90	1.50	1.50	0.02	56.8
N1-C8	1.359	2.18	−19.6	0.34	0.77	−1.40	1.22	3.86	2.51	1.72	0.05	75.1
C5-C6	1.420	2.05	−22.2	0.20	0.32	−1.08	1.23	7.68	2.79	1.87	0.04	53.5
C7-C6	1.458	1.92	−19.8	0.14	0.27	−0.99	1.07	5.57	2.46	1.95	0.03	53.2
C8-C7	1.423	2.07	−22.6	0.22	0.31	−1.07	1.14	10.21	3.16	1.90	0.01	56.7
C8-C9	1.508	1.74	−16.0	0.09	0.24	−0.88	0.95	3.51	2.16	1.98	0.02	52.4

^a^ *d*—bond length in Å; ^b^ electron density ρ_bcp_ in eÅ^−3^ and its corresponding Laplacian ∇^2^ρ_bcp_ in eÅ^−5^; ε—the bond ellipticity; *G*/ρ_bcp_ and *H*/ρ_bcp_—kinetic and total energy density over ρ_bcp_ ratios in he^−1^; Y_max_—ELI-D value at the attractor position; Δ_ELI_—the distance in Å of the attractor position perpendicular to the atom-atom axis; ^c^ δ—delocalization index; V_ELI_—is the volume of the ELI-D basin in Å^3^ cut at 0.001 au; ELI_pop_—the electron population within the ELI-D basin in e; and *RJI*—the Raub-Jansen index in %; ^d^ the optimized geometry of complex **2** shows a high similarity of ligand **1** and **2**, thus topological and integrated parameters are presented for only one ligand as an example; more data are presented in Table S3 (see Supplementary Materials).

**Table 4:** Cytotoxic activity of **1**, **2** and cisplatin (CDDP) towards human cancer cell lines.

Cell Line	1 IC_50_ (µM)	1 15 µM (%) ^a^	2 15 µM (%) ^a^	CDDP 15 µM (%) ^a^	CDDP IC_50_ (µM)
A2780	57 ± 3	ni	54 ± 9 ^b^	<10 ^c^	0.6 ± 0.03
A2780cis	50 ± 6	ni	65 ± 3	65 ± 1	20.2 ± 0.7
A549	79 ± 13	ni	71 ± 13 ^b^	21 ± 1.5	3.9 ± 0.5

^a^ Percentage of live cells, relatively to the control of the solvent (DMSO) assumed as 100%, in cultures treated with **1**, **2** or CDDP at indicated concentrations for 72 h; ^b^ results significantly different between the cell lines (Wilcoxon-Cox rank sum test, *p* < 0.05 for two sided tests); ^c^ max. concentration tested was 10 µM; ni—no decrease in cell viability.

## Supplementary Materials

Supplementary materials can be accessed at: http://www.mdpi.com/1420-3049/21/4/455/s1.

## Abbreviations

The following abbreviations are used in this manuscript:

**1**	3-hydroxyiminoflavanone
**2**	*cis*-(bis)(3-nitrosoflavanone)palladium(II)
CDDP	*cis*-diaminnadichloridoplatinum(II) or cisplatin
DMF	*N*′-*N*′-dimethylformamide
DMSO	dimethylsulfoxide
ELI-D	Electron Localizability Indicator
HAR	Hirshfeld Atom Refinement
IAM	Independent Atom Model
MTT	3-(4,5-dimethylthiazol-2-yl)-2,5-diphenyltetrazolium bromide
NAMI-A	New Anti-tumor Metastasis Inhibitor or imidazolium *trans*-DMSO-imidazole-tetrachlororuthenate(III)
NMR	Nuclear Magnetic Resonance
QTAIM	Quantum Theory of Atoms in Molecules
XWR	X-ray wavefunction refinement
