# Supplementary material for: New Look on 3-Hydroxyiminoflavanone and Its Palladium(II) Complex: Crystallographic and Spectroscopic Studies, Theoretical Calculations and Cytotoxic Activity
Source: Molecules. 2016 Apr 13;21(4):455. doi: 10.3390/molecules21040455 (PMC6273555; doi:10.3390/molecules21040455)
Supplement: Supplementary file 1 [file molecules-21-00455-s001.pdf]

Supplementary Materials: New Look on 3-Hydroxyiminoflavanone and Its Palladium(II) Complex: Crystallographic and Spectroscopic Studies, Theoretical Calculations and Cytotoxic Activity

Maria Kasprzak, Małgorzata Fabijańska, Lilianna Chęcińska, Leszek Szmigiero and Justyn Ochocki

Contents:

Figure S1. Residual density and deformation density maps of (**1**) in the plane of C1/C6/C9 atoms after the X-ray constrained wavefuction procedure (XCW).

Figure S2. (**a**) ^{1}H-NMR spectra of (**1**) (3-hydroxyiminoflavanone) in $CDCl_3$, taken at indicated times. The signal pattern shows the formation of two isomers; (**b**) the same spectra as above, only extended for better resolution; (**c**) the extended fragment of the spectra of (**1**) taken in $CDCl_3$ immediately after dissolution and after 144 h (six days), compared with the spectrum recorded in DMSO-d6.

Figure S3. ^{1}H-NMR spectrum of (**1**) (3-hydroxyiminoflavanone) in $CDCl_3$, taken immediately after dissolution.

Figure S4. UV-Vis spectra of the complex **2**, 15 μM in water and 0.2% *v*/*v* DMSO, collected at 37 °C, up to 62 h.

Figure S5. ^{13}C-NMR spectra of **1** in DMSO-d6 (the upper spectrum) and in $CDCl_3$ (the lower spectrum).

Table S1. Comparison of selected bond lengths and valence angles determined for experimental and theoretical models of (**1**) and (**2**).

Table S2. Properties integrated for (non-bonding) ELI-D lone-pair basins in XWR-model and OPT-model (**1**).

Table S3. Topological and integrated bond descriptors determined for selected bonds in OPT-model (**1**).

Table S4. Topological and integrated bond descriptors determined for selected bonds in OPT-model (**2**).

Table S5. Cartesian coordinates for the gas-phase structure of (**1**) (OPT-model) obtained at the BLYP/cc-pVTZ level of theory.

Table S6. Final positional parameters, anisotropic displacement parameters for non-hydrogen atoms and isotropic displacement parameters for hydrogen atoms obtained after Hirshfeld-atom refinement (HAR model) at the BLYP/cc-pVTZ level of theory for structure (**1**).

Table S7. Cartesian coordinates for the gas-phase structure of Pd-complex (**2**) (OPT-model) obtained at the BLYP/cc-pVTZ level of theory with the effective-core potential for Pd/ECP28MDF.

Figure S6. Cell viability after 72 h of exposure to **1**, **2** and the reference compound CDDP. All compounds were dissolved in DMSO. The results are displayed as mean ± SD.

Figure S7. ^{13}C-NMR spectrum of **1** in DMSO-d6 in the presence of $CDCl_3$.

Figure S8. ^{1}H-NMR spectrum of **1** in DMSO-d6 in the presence of $CDCl_3$.

Figure S9. ^{1}H-NMR spectrum of **1** in $CDCl_3$. The same sample as in Figure S7 and S8.

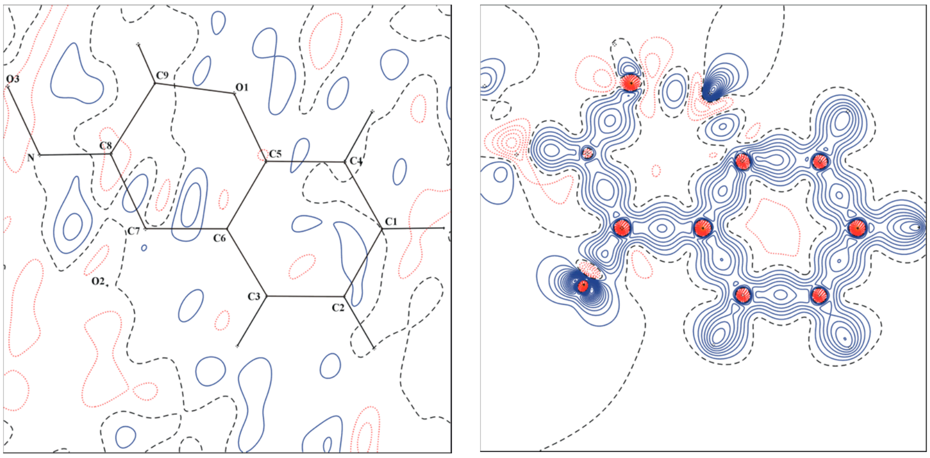

(**a**) (**b**)

Figure S1. Residual density (**a**) and deformation density (**b**) maps of **1** in the plane of C1/C6/C9 atoms after the X-ray constrained wavefuction procedure (XCW). Contour intervals are 0.05 eÅ^{-3} (**a**) and 0.1 eÅ^{-3} (**b**); positive, negative and zero contours are represented by solid blue, dotted red and dashed black lines, respectively.

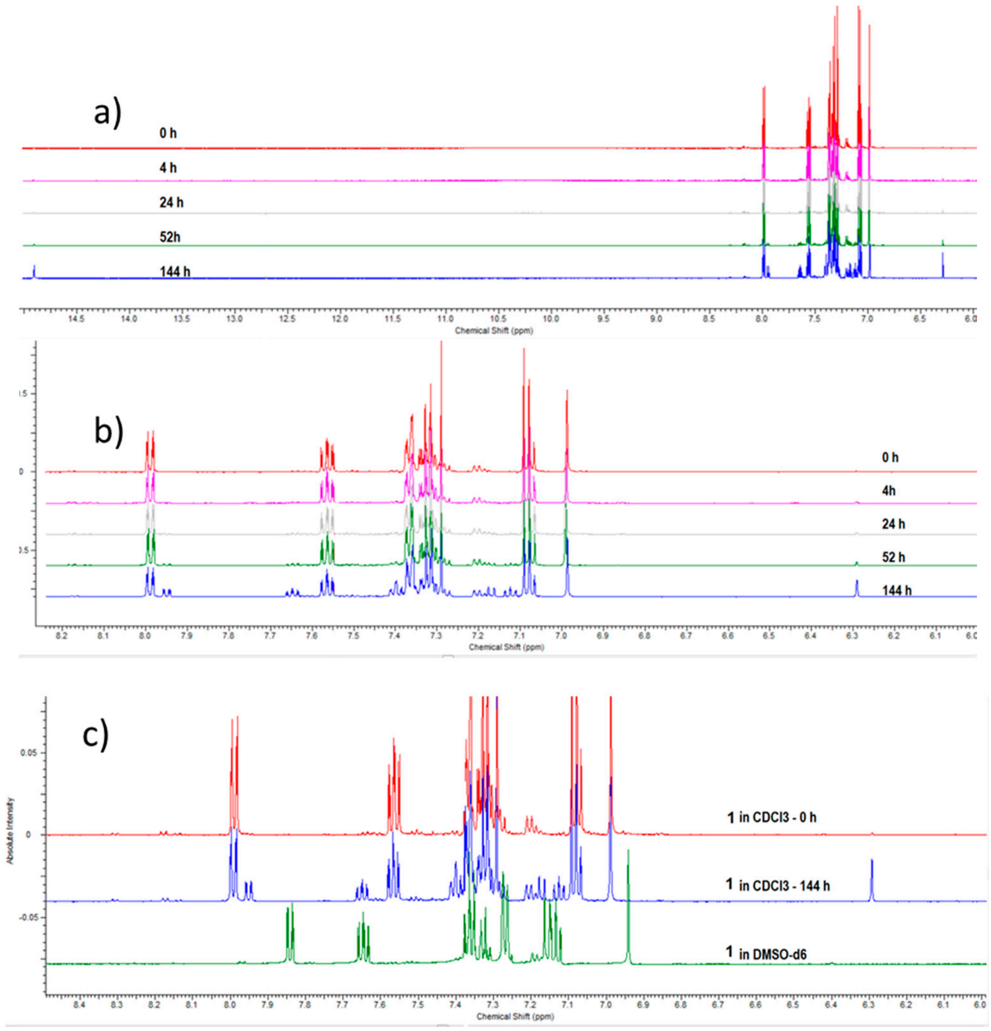

Figure S2. (**a**) ^{1}H-NMR spectra of **1** (3-hydroxyiminoflavanone) in $CDCl_3$, taken at indicated times. The signal pattern shows the formation of two isomers; (**b**) the same spectra as above, only extended for better resolution; (**c**) the extended fragment of the spectra of **1** taken in $CDCl_3$ immediately after dissolution and after 144 h (six days), compared with the spectrum recorded in DMSO-d_6.

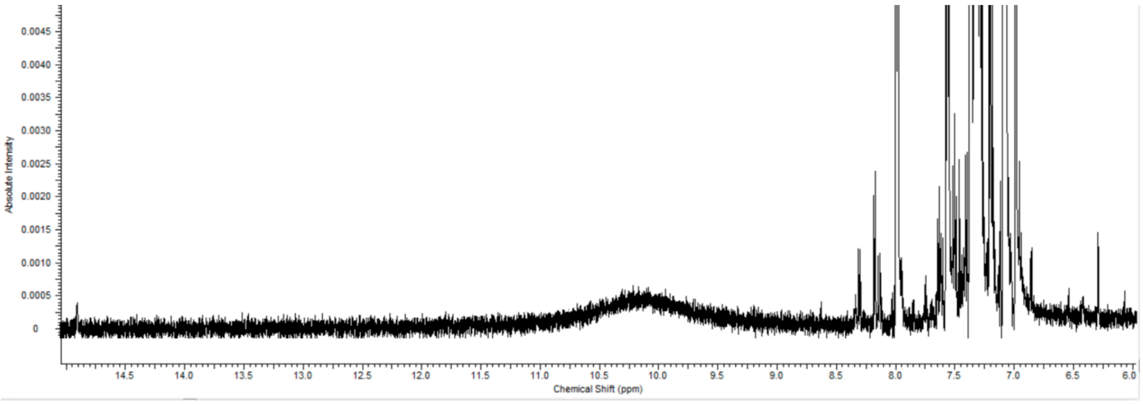

Figure S3. ^{1}H-NMR spectrum of **1** (3-hydroxyiminoflavanone) in $CDCl_3$, taken immediately after dissolution. The spectrum is extremely raised to show the very broad signal from the labile proton at approx. 10.15 ppm.

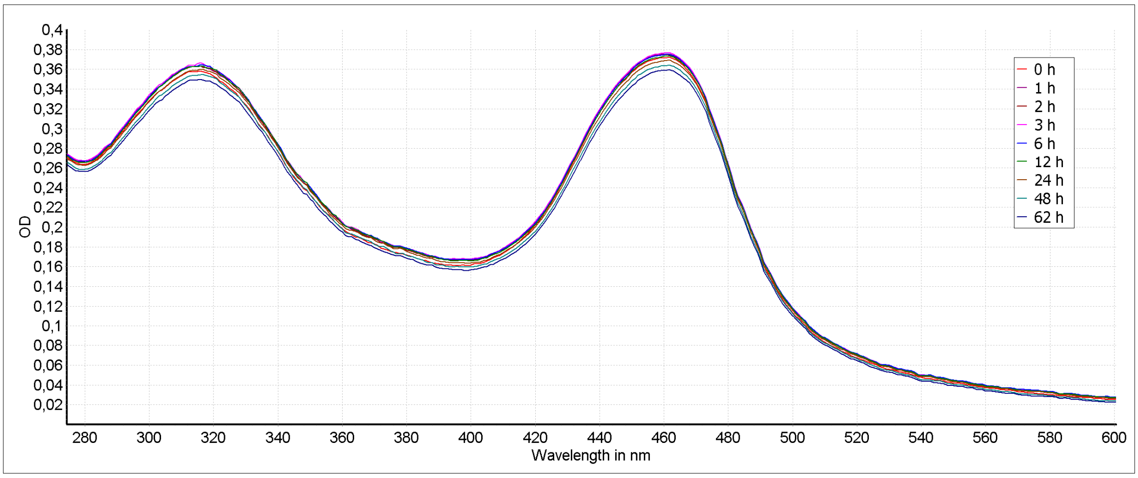

Figure S4. UV-Vis spectra of the complex **2**, 15 μM in water and 0.2% *v*/*v* DMSO, collected at 37 °C, up to 62 h.

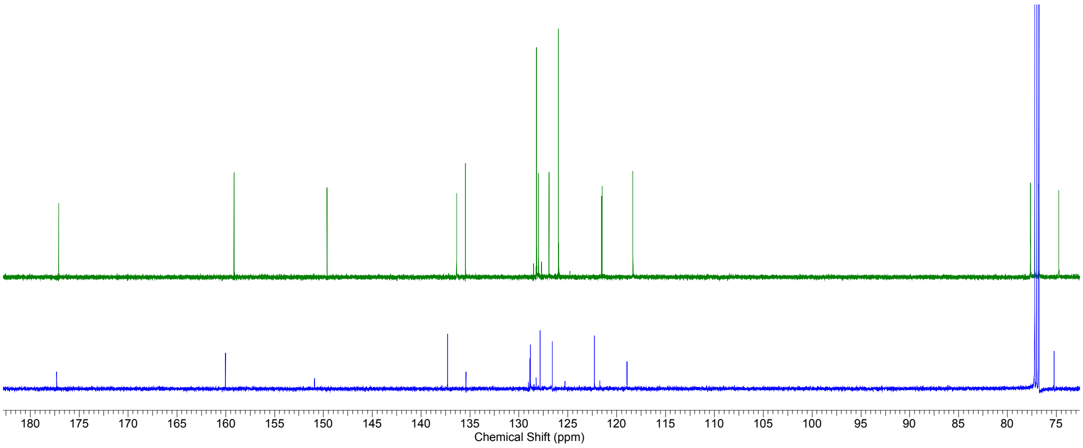

Figure S5. ^{13}C-NMR spectra of **1** in DMSO-d_6 (the upper spectrum) and in $CDCl_3$ (the lower spectrum).

Table S1. Comparison of selected bond lengths (Å) and valence angles (°) determined for experimental and theoretical models of **1** and **2**.

Bond/Angle	1			2	
	IAM	HAR	OPT	IAM	OPT
Pd1-N1	-	-	-	1.9898(16)	2.032
Pd1-N2	-	-	-	1.9809(16)	2.032
Pd1-O2	-	-	-	2.0586(13)	2.112
Pd1-O5	-	-	-	2.0636(13)	2.113
O2-Pd1-O5	-	-	-	97.42(5)	98.29
N1-Pd1-N2	-	-	-	99.92(6)	100.72
O2-Pd1-N1	-	-	-	81.25(6)	80.49
O2-Pd1-N2	-	-	-	178.50(6)	178.72
O5-Pd1-N1	-	-	-	177.54(6)	178.72
O5-Pd1-N2	-	-	-	81.45(6)	80.49
				**Ligand 1**	
O1-C5	1.3599(18)	1.3520(6)	1.372	1.357(2)	1.364
O1-C9	1.4468(17)	1.4405(6)	1.470	1.443(2)	1.485
O2-C7	1.2226(18)	1.2218(5)	1.229	1.272(2)	1.286
O3-N1	1.3757(18)	1.3641(5)	1.421	1.258(2)	1.249
N1-C8	1.285(2)	1.2856(6)	1.291	1.335(2)	1.359
C5-C6	1.400(2)	1.4026(6)	1.414	1.404(3)	1.420
C6-C7	1.465(2)	1.4614(7)	1.482	1.450(2)	1.458
C7-C8	1.484(2)	1.4839(7)	1.516	1.419(3)	1.423
C8-C9	1.503(2)	1.5015(7)	1.517	1.506(3)	1.508
O1-C9-C8	113.69(12)	113.69(8)	111.09	110.91(15)	110.91
O1-C9-C10	108.87(11)	109.07(7)	110.19	110.64(15)	108.84
C5-O1-C9	121.22(11)	121.56(7)	117.99	119.35(14)	120.23
O2-C7-C6	124.01(15)	124.01(9)	123.52	122.14(17)	121.60
O2-C7-C8	121.05(15)	121.10(9)	122.44	121.05(16)	121.03
N1-C8-C9	123.57(14)	123.44(9)	125.07	122.45(16)	120.77
N1-C8-C7	113.95(13)	114.19(8)	116.42	114.73(17)	115.69
O3-N1-C8	113.95(13)	114.58(8)	112.20	120.98(16)	121.13
				**Ligand 2**	
O4-C20	-	-	-	1.373(2)	1.364
O4-C24	-	-	-	1.447(2)	1.485
O5-C22	-	-	-	1.271(2)	1.286
O6-N2	-	-	-	1.259(2)	1.249
N1-C23	-	-	-	1.339(2)	1.359
C20-C21	-	-	-	1.399(3)	1.420
C21-C22	-	-	-	1.448(3)	1.458
C22-C23	-	-	-	1.419(3)	1.423
C23-C24	-	-	-	1.502(2)	1.508
O4-C24-C23	-	-	-	111.59(15)	110.91
O4-C24-C25	-	-	-	109.91(14)	108.84
C20-O4-C24	-	-	-	120.37(14)	120.23
O5-C22-C23	-	-	-	120.84(17)	121.02
N2-C23-C24	-	-	-	121.49(16)	120.77
N2-C23-C22	-	-	-	114.98(16)	115.69
O6-N2-C23	-	-	-	120.35(16)	121.13

IAM—independent atom model obtained from spherical refinement using SHELXL-2013; HAR—aspherical model obtained from Hirshfeld-atom refinement at blyp/cc-pVTZ; OPT—theoretical model obtained after full geometry optimization using Gaussian09 at blyp/cc-pVTZ.

Table S2. Properties integrated for (non-bonding) ELI-D lone pair basins in XWR-model (**1**) and OPT-model (**2**).

Lone Pair	1					2				
	V_{ELI}	ELI_{pop}	ELI_{max}	*RJI* (*e*)	*RJI* (%)	V_{ELI}	ELI_{pop}	Y_{max}	*RJI* (*e*)	*RJI* (%)
LP-O1	3.68	1.62	1.68	1.62	100	4.97	1.93	1.70	1.93	100
LP-O1	8.34	3.08	1.76	3.08	100	7.75	2.81	1.74	2.81	100
LP-O2	8.37	2.54	1.75	2.54	99.9	12.75	3.56	1.72	3.56	100
LP-O2	8.63	2.70	1.73	2.70	99.9	-	-	-	-	-
LP-O3	4.83	1.83	1.71	1.83	99.9	8.42	2.75	1.75	2.74	99.7
LP-O3	8.13	2.85	1.74	2.85	99.9	8.98	2.87	1.78	2.87	99.9
LP-N1	11.94	2.80	2.09	2.78	99.5	-	-	-	-	-
LP-O4	-	-	-	-	-	4.97	1.93	1.70	1.93	100
LP-O4	-	-	-	-	-	7.75	2.81	1.74	2.81	100
LP-O5	-	-	-	-	-	12.75	3.56	1.72	3.56	100
LP-O6	-	-	-	-	-	8.42	2.75	1.75	2.74	99.7
LP-O6	-	-	-	-	-	8.88	2.87	1.78	2.87	99.9
LP-Pd1	-	-	-	-	-	4.24	3.93	1.55	3.93	99.9
LP-Pd1	-	-	-	-	-	4.24	3.93	1.55	3.03	99.9
LP-Pd1	-	-	-	-	-	6.62	4.44	1.53	4.44	100
LP-Pd1	-	-	-	-	-	8.11	4.52	1.54	4.51	99.8

Table S3. Topological [a] and integrated [b] bond descriptors determined for selected bonds in OPT-model of **1**.

Bond	ρ_{bcp}	$\nabla^2\rho_{bcp}$	ε	G/ρ_{bcp}	H/ρ_{bcp}	δ	V_{ELI}	ELI_{pop}	Y_{max}	Δ_{ELI}	*RJI*
O1-C5	1.94	−15.0	0.06	0.87	−1.41	0.95	1.21	1.45	1.59	0.04	76.3
O1-C9	1.57	−12.2	0.03	0.63	−1.18	0.85	1.07	1.24	1.56	0.05	75.8
O2-C7	2.73	−11.4	0.09	1.41	−1.70	1.42	4.16	2.18	1.54	0.01	71.3
O3-N1	1.99	−3.7	0.05	0.62	−0.75	1.26	0.57	0.93	1.44	0.03	54.1
N1-C8	2.55	−21.3	0.29	0.99	−1.58	1.54	6.63	3.02	1.71	0.08	68.3
C5-C6	2.08	−22.7	0.20	0.33	−1.09	1.25	8.06	2.83	1.86	0.03	54.0
C6-C7	1.84	−17.8	0.13	0.94	−0.26	1.03	5.42	2.53	1.94	0.01	50.6
C8-C7	1.73	−15.5	0.11	0.24	−0.87	0.92	5.07	2.37	1.96	0.02	46.3
C9-C8	1.72	−15.3	0.05	0.24	−0.86	0.93	3.58	2.15	1.97	0.01	52.7

[a] electron density ρ_{bcp} in eÅ^{-3} and its corresponding Laplacian $\nabla^2\rho_{bcp}$ in eÅ^{-5}; ε—the bond ellipticity; G/ρ_{bcp} and H/ρ_{bcp}—kinetic and total energy density over ρ_{bcp} ratios in he^{-1}; Y_{max}—ELI-D value at the attractor position; Δ_{ELI}—the distance in Å of the attractor position perpendicular to the atom-atom axis; [b] δ—delocalization index; V_{ELI}—is the volume of the ELI-D basin in Å^3 cut at 0.001 au; ELI_{pop}—the electron population within the ELI-D basin in *e*; and *RJI*—the Raub-Jansen index in %.

Table S4. Topological [a] and integrated [b] bond descriptors determined for selected bonds in OPT-model of **2**.

Bond	*d*	ρ_{bcp}	$\nabla^2\rho_{bcp}$	ε	G/ρ_{bcp}	H/ρ_{bcp}	δ	V_{ELI}	ELI_{pop}	Υ_{max}	Δ_{ELI}	*RJI*
N1-Pd1	2.032	0.80	8.2	0.07	1.07	−0.36	0.76	6.42	2.53	1.77	0.09	94.5
N2-Pd1	2.032	0.80	8.2	0.07	1.07	−0.36	0.76	6.42	2.53	1.77	0.09	94.5
O2-Pd1	2.112	0.57	8.6	0.04	1.27	−0.22	0.56	2.80	2.02	1.61	0.03	97.1
O5-Pd1	2.113	0.57	8.6	0.04	1.27	−0.22	0.56	2.80	2.02	1.61	0.03	97.1
O1-C5	1.364	1.97	−14.1	0.05	0.95	−1.45	0.97	1.23	1.47	1.59	0.05	76.1
O1-C9	1.485	1.51	−11.3	0.03	0.61	−1.14	0.83	1.10	1.24	1.56	0.06	76.4
O2-C7	1.286	2.40	−15.5	0.07	1.14	−1.60	1.19	1.94	1.82	1.57	0.01	73.7
N1-O3	1.249	3.15	−21.7	0.06	0.74	−1.22	1.66	0.90	1.50	1.50	0.02	56.8
N1-C8	1.359	2.18	−19.6	0.34	0.77	−1.40	1.22	3.86	2.51	1.72	0.05	75.1
C5-C6	1.420	2.05	−22.2	0.20	0.32	−1.08	1.23	7.68	2.79	1.87	0.04	53.5
C7-C6	1.458	1.92	−19.8	0.14	0.27	−0.99	1.07	5.57	2.46	1.95	0.03	53.2
C8-C7	1.423	2.07	−22.6	0.22	0.31	−1.07	1.14	10.21	3.16	1.90	0.01	56.7
C8-C9	1.508	1.74	−16.0	0.09	0.24	−0.88	0.95	3.51	2.16	1.98	0.02	52.4
O4-C20	1.364	1.97	−14.1	0.05	0.95	−1.45	0.97	1.23	1.47	1.59	0.05	76.1
O4-C24	1.485	1.51	−11.3	0.03	0.61	−1.14	0.83	1.10	1.24	1.56	0.06	76.4
O5-C22	1.286	2.40	−15.5	0.07	1.14	−1.60	1.19	1.94	1.82	1.57	0.01	73.7
O6-N2	1.249	3.15	−21.7	0.06	0.74	−1.22	1.66	1.90	1.50	1.51	0.02	56.8
N2-C23	1.359	2.18	−19.6	0.34	0.77	−1.40	1.22	3.86	2.51	1.72	0.05	75.1
C20-C21	1.420	2.05	−22.2	0.20	0.32	−1.08	1.23	7.68	2.79	1.87	0.04	53.5
C22-C21	1.458	1.92	−19.8	0.14	0.27	−0.99	1.07	5.57	2.46	1.95	0.03	53.2
C23-C22	1.423	2.07	−22.6	0.22	0.31	−1.07	1.14	10.21	3.16	1.90	0.01	56.7
C23-C24	1.508	1.74	−16.0	0.09	0.24	−0.88	0.95	3.51	2.16	1.98	0.02	52.4

[a] electron density ρ_{bcp} in eÅ^{-3} and its corresponding Laplacian $\nabla^2\rho_{bcp}$ in eÅ^{-5}; ε—the bond ellipticity; G/ρ_{bcp} and H/ρ_{bcp}—kinetic and total energy density over ρ_{bcp} ratios in he^{-1}; Υ_{max}—ELI-D value at the attractor position; Δ_{ELI}—the distance in Å of the attractor position perpendicular to the atom-atom axis; [b] δ—delocalization index; V_{ELI}—is the volume of the ELI-D basin in Å^3 cut at 0.001 au; ELI_{pop}—the electron population within the ELI-D basin in *e*; and *RJI*—the Raub-Jansen index in %.

Table S5. Cartesian coordinates (X, Y, Z in Å) for the gas-phase structure of ligand **1** (OPT-model) obtained at the BLYP/cc-pVTZ level of theory.

O	−0.475412	−0.312214	−1.576952
O	−1.385144	2.280553	1.536758
O	1.714234	2.978668	−1.173344
N	0.581865	2.892627	−0.318625
C	−3.459571	−1.961754	−0.293513
C	−3.791930	−1.164111	0.818163
C	−3.004837	−0.062817	1.128876
C	−2.343933	−1.668366	−1.073822
C	−1.542143	−0.560876	−0.750364
C	−1.872532	0.262588	0.350799
C	−1.094558	1.489324	0.642428
C	0.094883	1.698262	−0.273159
C	0.624667	0.510429	−1.054710
C	1.618125	−0.365524	−0.275391
C	2.190959	0.049830	0.935431
C	3.121125	−0.762882	1.596374
C	3.487023	−1.997960	1.054886
C	2.921662	−2.417385	−0.156538
C	1.996939	−1.606256	−0.818578
H	1.917890	3.930124	−1.131096
H	−4.073955	−2.824080	−0.545399
H	−4.661592	−1.406666	1.423975
H	−3.240779	0.586929	1.968139
H	−2.071887	−2.281057	−1.929513
H	1.111032	0.877244	−1.964220
H	1.919330	1.009359	1.368513
H	3.553829	−0.428011	2.536903
H	4.207229	−2.630237	1.570127
H	3.203525	−3.376232	−0.587255
H	1.557623	−1.936841	−1.756784

Table S6. Final positional parameters (in Å), anisotropic displacement parameters (in Å²) for non-hydrogen atoms and isotropic displacement parameters (in Å²) for hydrogen atoms obtained after Hirshfeld-atom refinement (HAR model) at the BLYP/cc-pVTZ level of theory for structure **1**.

O1	0.07298(8)	0.2296(1)	0.11857(3)	0.017(1)	0.022(1)	0.025(1)	−0.007(1)	0.002(1)	0.001(1)
O2	0.18491(9)	0.7913(1)	0.02083(3)	0.028(2)	0.026(1)	0.021(1)	−0.008(1)	0.005(1)	0.002(1)
O3	0.48299(8)	0.2599(1)	0.04306(3)	0.020(1)	0.034(1)	0.019(1)	−0.002(1)	0.003(1)	−0.001(1)
N	0.3835(1)	0.4420(1)	0.03393(3)	0.018(2)	0.033(2)	0.014(1)	−0.006(1)	0.003(1)	−0.002(1)
C1	−0.2809(1)	0.5579(2)	0.12515(4)	0.018(2)	0.030(2)	0.023(2)	−0.004(2)	0.000(2)	−0.005(2)
C2	−0.2521(1)	0.7561(2)	0.09328(4)	0.020(2)	0.029(2)	0.023(2)	−0.001(2)	−0.002(1)	−0.005(2)
C3	−0.1103(1)	0.7793(2)	0.06987(4)	0.023(2)	0.023(2)	0.017(2)	−0.004(2)	−0.002(1)	−0.002(1)
C4	−0.1703(1)	0.3860(2)	0.13324(4)	0.017(2)	0.026(2)	0.021(2)	−0.007(2)	0.001(1)	−0.001(2)
C5	−0.0266(1)	0.4091(2)	0.10964(4)	0.016(2)	0.021(2)	0.016(2)	−0.007(1)	0.001(1)	−0.003(1)
C6	0.0043(1)	0.6069(2)	0.07763(4)	0.019(2)	0.022(2)	0.014(2)	−0.006(1)	0.000(1)	−0.003(1)
C7	0.1519(1)	0.6287(2)	0.05103(4)	0.021(2)	0.022(2)	0.015(2)	−0.007(1)	0.002(1)	−0.002(1)
C8	0.2619(1)	0.4338(2)	0.06263(4)	0.018(2)	0.025(2)	0.013(2)	−0.007(1)	0.002(1)	−0.003(1)
C9	0.2327(1)	0.2452(2)	0.10462(4)	0.018(2)	0.019(2)	0.019(2)	−0.005(1)	0.002(1)	−0.004(1)
C10	0.3356(1)	0.2729(1)	0.15914(4)	0.015(2)	0.015(2)	0.017(2)	−0.001(1)	0.003(1)	−0.002(1)
C11	0.3239(1)	0.4691(2)	0.19324(4)	0.016(2)	0.019(2)	0.016(2)	0.001(1)	0.000(1)	−0.003(1)
C12	0.4168(1)	0.4920(2)	0.24366(4)	0.021(2)	0.024(2)	0.016(2)	0.000(1)	0.001(1)	−0.003(1)
C13	0.5226(1)	0.3194(2)	0.26030(4)	0.022(2)	0.031(2)	0.018(2)	−0.001(2)	−0.002(2)	0.005(2)
C14	0.5357(1)	0.1239(2)	0.22653(4)	0.023(2)	0.024(2)	0.027(2)	0.006(2)	0.002(2)	0.008(2)
C15	0.4421(1)	0.1008(2)	0.17608(4)	0.022(2)	0.016(2)	0.024(2)	0.003(1)	0.005(2)	0.000(1)
H1	0.566(2)	0.306(2)	0.0172(6)	0.04(1)	0.04(1)	0.04(1)			
H2	−0.394(1)	0.534(2)	0.1435(5)	0.04(1)	0.04(1)	0.04(1)			
H3	−0.341(1)	0.888(2)	0.0866(5)	0.05(1)	0.05(1)	0.05(1)			
H4	−0.087(1)	0.925(2)	0.0456(5)	0.04(1)	0.04(1)	0.04(1)			
H5	−0.193(1)	0.234(2)	0.1550(5)	0.04(1)	0.04(1)	0.04(1)			
H6	0.259(1)	0.081(2)	0.0865(5)	0.04(1)	0.04(1)	0.04(1)			
H7	0.242(1)	0.599(2)	0.1801(5)	0.04(1)	0.04(1)	0.04(1)			
H8	0.405(1)	0.642(2)	0.2690(5)	0.04(1)	0.04(1)	0.04(1)			
H9	0.592(1)	0.334(2)	0.2990(5)	0.05(1)	0.05(1)	0.05(1)			
H10	0.615(1)	−0.012(2)	0.2397(5)	0.04(1)	0.04(1)	0.04(1)			
H11	0.450(1)	−0.047(2)	0.1486(5)	0.04(1)	0.04(1)	0.04(1)			

Table S7. Cartesian coordinates (X, Y, Z in Å) for the gas-phase structure of Pd-complex **2** (OPT-model) obtained at the BLYP/cc-pVTZ level of theory with the effective-core potential for Pd/ECP28MDF at the cc-pVTZ (initial geometry of structure **2** was taken from the corresponding crystal structure).

Pd	−0.000007	−0.579330	−0.360119
O	5.183046	−0.249915	−1.113319
O	1.597860	−1.807659	0.272837
O	1.488278	1.678546	−1.528558
O	−5.183122	−0.249877	−1.113157
O	−1.597873	−1.807675	0.272855
O	−1.488329	1.678559	−1.528499
N	1.564542	0.566143	−0.966756
N	−1.564578	0.566136	−0.966726
C	6.451608	−3.244049	0.519130
H	7.408540	−3.747219	0.643714
C	5.289325	−3.799889	1.087662
H	5.346239	−4.729449	1.648332
C	4.070800	−3.153765	0.922057
H	3.153702	−3.564114	1.336483
C	6.399690	−2.051456	−0.198703
H	7.291204	−1.613653	−0.639859
C	5.171280	−1.386841	−0.359434
C	3.990019	−1.943377	0.199236
C	2.720752	−1.269458	−0.048895
C	2.768816	0.001932	−0.685648
C	4.052982	0.709784	−1.036286
H	3.974579	1.100575	−2.057330
C	4.445992	1.861346	−0.114267
C	4.255490	1.800516	1.276025
H	3.769312	0.936386	1.723252
C	4.678429	2.851914	2.095565
H	4.523551	2.793693	3.171105
C	5.290086	3.979235	1.534987
H	5.612297	4.799558	2.173202
C	5.476649	4.051443	0.149717
H	5.942432	4.929010	−0.294106
C	5.057112	2.997722	−0.667508
H	5.197812	3.058472	−1.745582
C	−6.451604	−3.244116	0.519137
H	−7.408531	−3.747295	0.643728
C	−5.289295	−3.799999	1.087583
H	−5.346189	−4.729601	1.648186
C	−4.070781	−3.153862	0.921976
H	−3.153665	−3.564232	1.336340
C	−6.399715	−2.051477	−0.198614
H	−7.291248	−1.613639	−0.639701
C	−5.171307	−1.386850	−0.359360
C	−3.990028	−1.943420	0.199233
C	−2.720770	−1.269485	−0.048878
C	−2.768841	0.001916	−0.685616
C	−4.052998	0.709790	−1.036228
H	−3.974639	1.100520	−2.057297
C	−4.445955	1.861396	−0.114248
C	−4.255247	1.800689	1.276021
H	−3.768908	0.936645	1.723244
C	−4.678176	2.852106	2.095543
H	−4.523138	2.793981	3.171064
C	−5.290026	3.979323	1.534967
H	−5.612235	4.799659	2.173166
C	−5.476790	4.051409	0.149716
H	−5.942721	4.928898	−0.294105
C	−5.057260	2.997671	−0.667488
H	−5.198112	3.058326	−1.745550

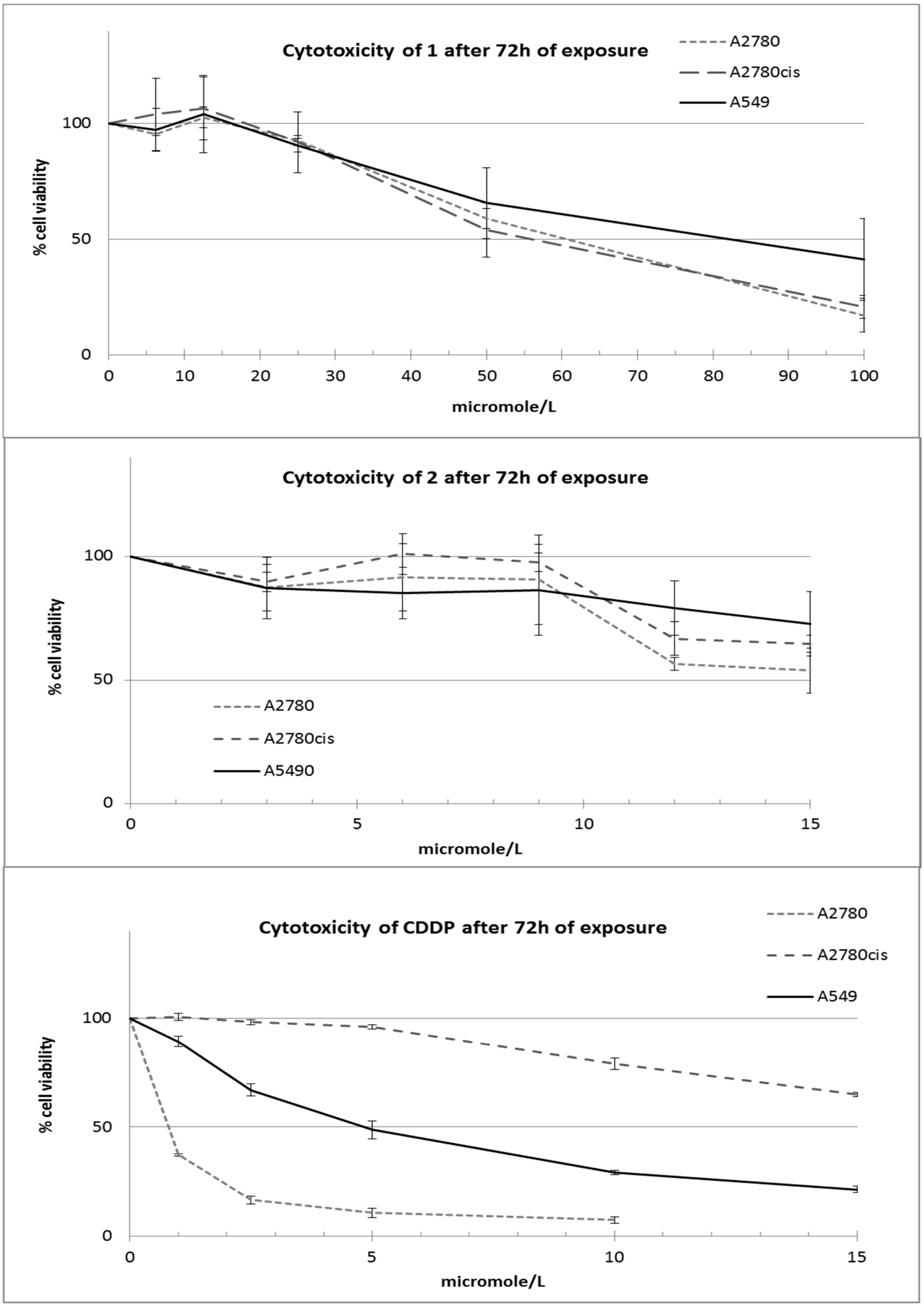

Figure S6. Cell viability after 72 h of exposure to **1**, **2** and the reference compound CDDP. All compounds were dissolved in DMSO. The results are displayed as mean ± SD.

In the included 13C-NMR spectrum of **1** in DMSO-d6 there is a triplet around 77 ppm, characteristic for chloroform. It is a result of the presence of chloroform presence in the sample. The spectrum was taken during our first experiments, on older 300 MHz apparatus. A portion of 1 was dissolved in $CDCl_3$ and resulting spectrum was of illegible, because of relatively poor solubility of **1**

in chloroform. Then we added DMSO-d6 and more of **1** to the same sample, and then recorded the spectrum again. Anyway, we decided to use the spectrum for interpretation, because 1H-NMR spectrum of the same sample shows the signal of the oxime proton. Therefore, we assume that the isomeric oxime form of 3-HIF is not affected by the presence of chloroform residues. The ^{13}C and ^{1}H-NMR spectra of the same sample are given below for comparison.

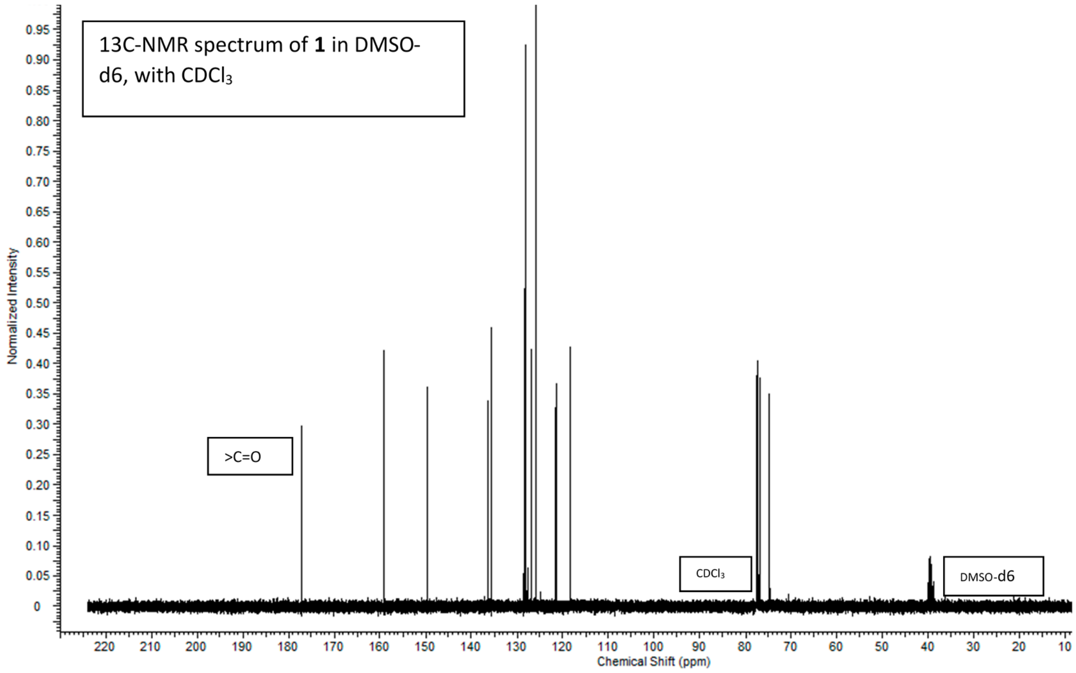

Figure S7. ^{13}C-NMR spectrum of **1** in DMSO-d6 in the presence of $CDCl_3$.

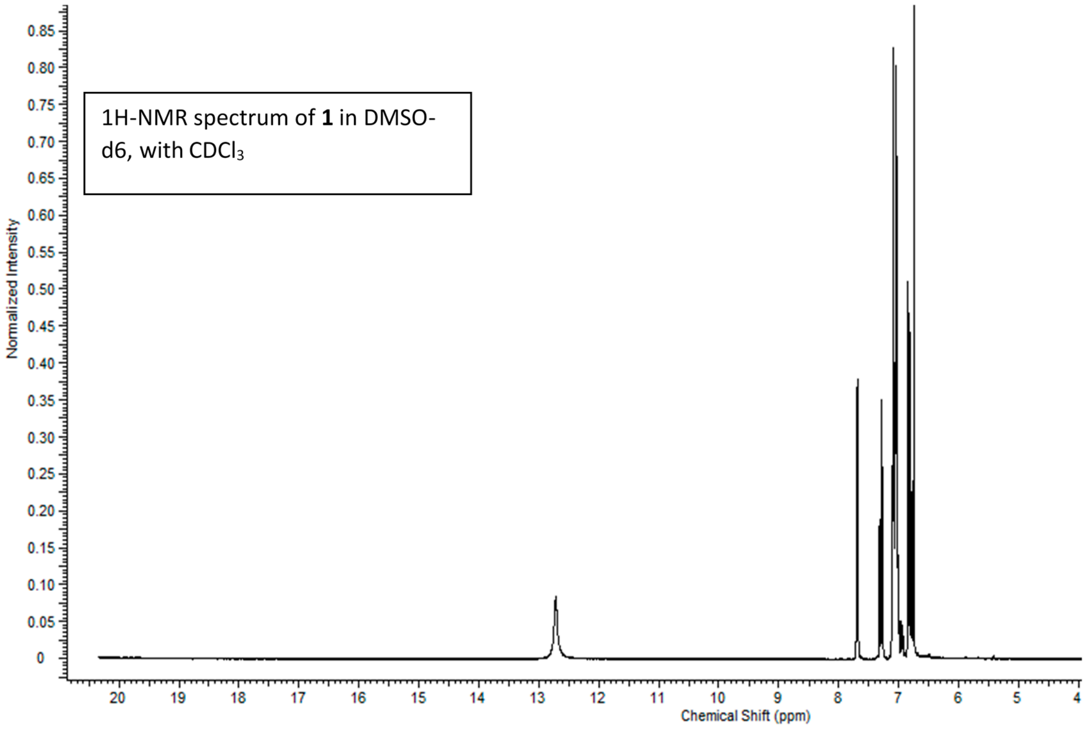

Figure S8. ^{1}H-NMR spectrum of 1 in DMSO-d6 in the presence of $CDCl_3$.

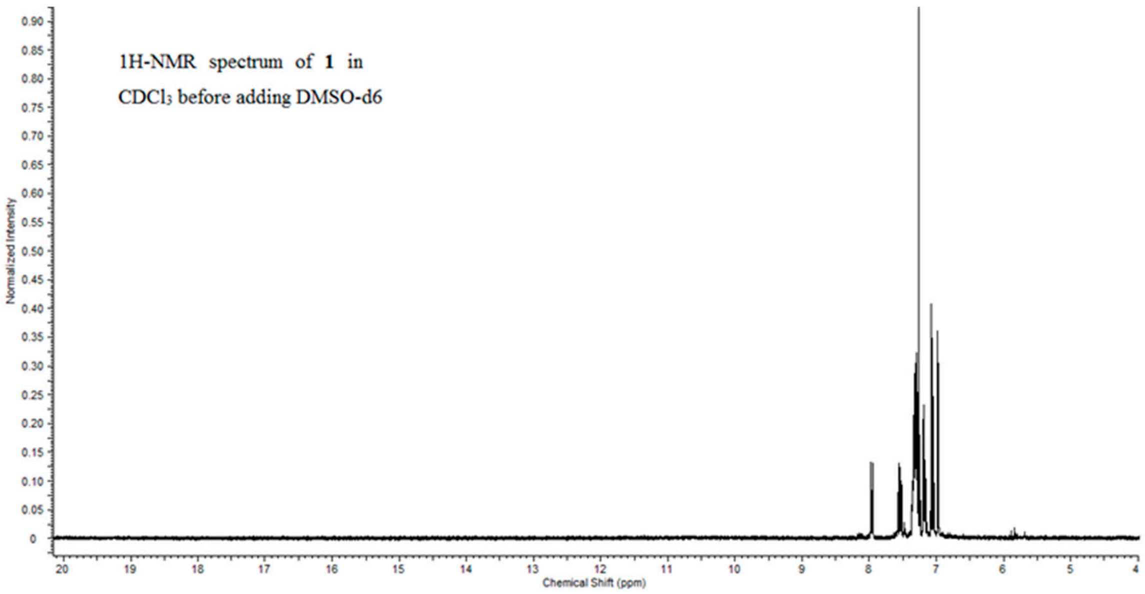

Figure S9. ^{1}H-NMR spectrum of 1 in $CDCl_3$. The same sample as in Fig. S7 and S8.
